# A higher-order finite element reactive transport model for unstructured and fractured grids

**DOI:** 10.1038/s41598-020-72354-3

**Published:** 2020-09-23

**Authors:** Joachim Moortgat, Mengnan Li, Mohammad Amin Amooie, Di Zhu

**Affiliations:** 1 School of Earth Sciences, The Ohio State University, Columbus, OH 43210 USA; 2 Department of Chemical Engineering, Massachusetts Institute of Technology, Cambridge, MA 02139 USA; 3 Occidental Petroleum Corporation, Houston, TX 77046 USA

**Keywords:** Biogeochemistry, Hydrology

## Abstract

This work presents a new reactive transport framework that combines a powerful geochemistry engine with advanced numerical methods for flow and transport in subsurface fractured porous media. Specifically, the PhreeqcRM interface (developed by the USGS) is used to take advantage of a large library of equilibrium and kinetic aqueous and fluid-rock reactions, which has been validated by numerous experiments and benchmark studies. Fluid flow is modeled by the Mixed Hybrid Finite Element (FE) method, which provides smooth velocity fields even in highly heterogenous formations with discrete fractures. A multilinear Discontinuous Galerkin FE method is used to solve the multicomponent transport problem. This method is locally mass conserving and its second order convergence significantly reduces numerical dispersion. In terms of thermodynamics, the aqueous phase is considered as a compressible fluid and its properties are derived from a Cubic Plus Association (CPA) equation of state. The new simulator is validated against several benchmark problems (involving, e.g., Fickian and Nernst-Planck diffusion, isotope fractionation, advection-dispersion transport, and rock-fluid reactions) before demonstrating the expanded capabilities offered by the underlying FE foundation, such as high computational efficiency, parallelizability, low numerical dispersion, unstructured 3D gridding, and discrete fraction modeling.

## Introduction

The past decades have seen a recognition of the importance of (geo-) chemical and (micro-) biological reactions in the subsurface environment and how those reactions are intricately coupled to fluid flow and even the geomechanical properties of the host medium. As two examples: (1) fluid flow paths in heterogeneous porous media determine what rock minerals encounter what fluid compositions and thus affect the degree of rock-fluid reactions , which may differ from (smaller and more homogeneous) batch reaction experiments, and (2) rock dissolution and precipitation due to geochemical reactions can locally change the porosity and permeability of a rock matrix as well as fracture apertures and thus impact fluid flow.

As a result of the aforementioned, interest is growing in multiphysics simulators that can simultaneously model a wide variety of coupled processes. As the topic of interest in this work, geochemistry initially used to be modeled mostly as a local batch-reactor process with limited to no transport modeling^1,2^. However, ever more physics has been included in more recent codes such as PHT3D^3^, HPx^4^, and OpenGeoSys^5^, which all use Phreeqc^6^ as the geochemistry engine. Other codes with native geochemistry include CrunchFlow^7–9^, PFLOTRAN^10^, TOUGHREACT^11,12^, ORCHESTRA^13^, eSTOMP^14^, MIN3P^15^, and HYDROGEOCHEM^16^. This list is not exhaustive but includes the most widely used reactive transport models that were, moreover, compared in a comprehensive benchmarking study^17,18^. Most of these codes now allow for three-dimensional problems, four allow for multiphase and variable density flow, and three use continuous Galerkin FE methods that allow for unstructured grids.

In this work, we build on those achievements to include further capabilities that were initially developed primarily for hydrocarbon (oil and gas) reservoirs and have not been used in reactive transport modeling of hydrogeology problems. Specifically, the transport of water, hydrocarbons, nitrogen, carbon dioxide, tracers, and any dissolved chemically reactive species is updated with a higher-order Discontinuous Galerkin (DG) FE method^19,20^. Powerful features of this method are that it provides strict local mass conservation at the grid-cell level, it is massively parallelizable, the discontinuous formulation is a natural choice for heterogenous layered and fractured formations, and finally it has low numerical dispersion^21^.

The flow problem is discretized by a Mixed Hybrid Finite Element (MHFE) method^22^, which simultaneously (and to the same order of accuracy) solves for globally continuous pressure and velocity fields. Mixed FE methods are known to have low grid sensitivity^20,23^. Their other main strength is to provide accurate velocity fields^24^, particularly for heterogeneous and fractured domains^25,26^. These features also allow for an efficient discrete fracture model based on cross-flow equilibrium^27–33^.

The combined DG and MHFE methods^34^ are implemented for any mixture of affine elements, i.e., triangles and quadrilaterals in two dimensions (2D) and hexahedra, tetrahedra, and prisms in 3D, which allows for natural discretization (gridding) of complex formation architectures. Moreover, these methods automatically allow for full permeability and dispersion tensors, unlike all but one of the aforementioned reactive transport models^16^. We do not use *adaptive*-mesh-refinement and the computational cost of constructing unstructured grids is negligible compared to the total simulation times.

To couple these well-established numerical methods for flow and transport to an equally mature geochemistry engine, we follow a similar approach as in^3–5,35^ and take advantage of the useful PhreeqcRM interface^36^. Stand-alone Phreeqc can model a wide range of equilibrium and kinetic reactions with results generally agreeing with the other reactive transport codes in the aforementioned benchmark study^17,18^. Its main limitation is its 1D transport model, but this was alleviated by the PhreeqcRM interface, which allows the full capabilities of Phreeqc to be efficiently coupled to any flow and transport simulator.

The ultimate goal of this and future work is to combine the full capabilities of Phreeqc with those of our in-house simulator, *Osures*, which in addition to the previously discussed finite element methods for flow and transport has several other features such as (1) a broad suite of thermodynamic phase stability and phase-split algorithms for multiphase multicomponent mixtures of water, oil (including several liquid hydrocarbon phases), gas (and supercritical fluids), and asphaltenes, (2) both Peng-Robinson^37^ and Cubic Plus Association (CPA) equations of state (EOS)^21,38^ with the latter improving the phase behavior calculations for the aqueous phase, (3) no limitations on compressibility and density changes, (4) composition dependent capillary pressures, (5) a thermodynamically consistent model for multiphase multicomponent Fickian diffusion that relies on a full matrix of composition-dependent diffusion coefficients^30,39,40^. This work presents the first step towards this goal, in which only an aqueous phase is considered, but allowing for compressibility and density changes.

The following sections first discuss implementation details of this new reactive transport model before presenting a range of numerical experiments to validate this approach and demonstrate its novel features and strengths.

## Formulation

### Flow and transport

In this section the governing equations are provided in a general multiphase and multicomponent formulation in which all phases are treated equally (e.g., allowing for compressibility and density changes).

The transport equations are written in terms of molar conservation of each component *i* out of $n_{c}$ total number of components, including all reacting and non-reacting components (defined in more detail in the next subsection):

1 $$\begin{aligned} \phi \frac{\partial c_i}{\partial t} + \nabla \cdot \vec {U}_i= & {} F^{{\mathrm {well}}}_i + F^{{\mathrm {react}}}_{i}, \quad i = 1, \ldots , n_c, \end{aligned}$$

with $\phi \ [\cdot ]$ the porosity, $c_i\ [{\mathrm {mol}}/{{\mathrm {m}}}^{3}]$ the molar density of component *i* (total molar density in the case of multiphase mixtures), $F^{{\mathrm {well}}}_i\ [{\mathrm {mol}}/({{\mathrm {s}}}\ {{\mathrm {m}}}^{3})]$ a source or sink of component *i* (e.g., a contaminant spill site or a way to prescribe inflow and outflow conditions), and $F^{\mathrm {react}}_i\ [{\mathrm {mol}}/({\mathrm {s}}\ {{\mathrm {m}}}^{3})]$ the source or sink of component *i* due to geochemical reactions.

The component flux $U_{i}$ contains both the advective and dispersive contributions. In the most general case of $n_{\mathrm {ph}}$ number of phases that are labeled by $\alpha = 1, \ldots , n_{\mathrm {ph}}$, $U_{i}$ is given by

2 $$\begin{aligned} \vec {U}_i= & {} \sum _{\alpha =1}^{n_{\mathrm {ph}}} \left( c_{i,\alpha } \vec {u}_\alpha + f(\phi ,\tau ) S_\alpha \vec {J}_{i,\alpha }\right) ,\quad i = 1, \ldots , n_c, \end{aligned}$$

with $c_{i,\alpha }\ [{\mathrm {mol}}/{{\mathrm {m}}}^{3}]$ the molar density of component *i* in phase $\alpha$, $\vec {u}_\alpha \ [{{\mathrm {m}}}/{\mathrm {s}}]$ the fiducial Darcy velocity

3 $$\begin{aligned} \vec {u}_\alpha= & {} - \lambda _{\alpha }{\mathrm {K}} (\nabla p_{\alpha } - \rho _\alpha \vec {g}), \quad \alpha = 1, \ldots , {n_{\mathrm {ph}}} \end{aligned}$$

in which $p_{\alpha }\ [{\mathrm {Pa}}]$ is the phase pressure, $\vec {g}$ is the gravitational vector, and $\lambda _{\alpha }\ [{{\mathrm {m}}}\ {\mathrm {s}}/{\mathrm {kg}}]= \lambda _\alpha (S_\alpha )$ is the phase mobility, $\rho _\alpha \ [{\mathrm {kg}}/{\mathrm {m}}^{3}]$ the phase mass density, $S_{\alpha }\ [\cdot ]$ the phase saturation, and $\mathrm {K}\ [{\mathrm {m}}^{2}]$ the full permeability tensor. The diffusive term $f(\phi ,\tau ) S_\alpha \vec {J}_{i,\alpha }$ is discussed in detail below.

For a fully compressible multiphase system, the pressure (of a reference phase) evolves as^41,42^:

4 $$\begin{aligned}&\phi C_{f} \frac{\partial p}{\partial t} + \sum _{i=1}^{n_c} {\overline{\nu }_i(\nabla \cdot \vec {U}_i}-F^{\mathrm {well}}_i - F^{\mathrm {react}}_{i}) =0, \end{aligned}$$

with $C_{f}\ [\mathrm {Pa}^{{-1}}]$ the total fluid compressibility of the multiphase mixture, and $\overline{\nu }_i\ [{\mathrm {m}}^{3}/{\mathrm {mol}}]$ the total partial molar volume of each component. The algorithm to compute these parameters for multiphase mixtures is highly non-linear^43^.

For the case of a single aqueous phase the expressions for compressibility and partial molar volumes are considerably simpler, and $n_{\mathrm {ph}} =1$, $\alpha = w$, $c_{i,\alpha } = c_{i}$, $\lambda _{\alpha } = \lambda _{w} = 1/\mu _{w}$ with $\mu _{w}\ [{\mathrm {m}}\ {\mathrm {s}}/{\mathrm {kg}}]$ the water viscosity, $S_{w}=1$, and $p_{\alpha }=p$ (no capillary effects).

### Geochemical reactions

When several species react through a number of different reactions, the concentrations of each of the species are not independent. For example, in the equilibrium reaction $\hbox {H}_{2}\hbox {O} \rightleftharpoons \hbox {H}^{+} + \hbox {OH}^{-}$, if one mole of $\hbox {H}_{2}\hbox {O}$ reacts, the increase in $\hbox {H}^{+}$ and $\hbox {OH}^{-}$ concentrations equals the decrease in $\hbox {H}_{2}\hbox {O}$ concentration. A mathematical consequence is that not all species concentrations need to be transported explicitly. One can split the total number of species into a subset of independent primary components and a set of secondary components that can be constructed from the primary ones^44^. The process has been described in the literature^18^ but is perhaps best illustrated by example.

Consider a typical mixture in the context of geological carbon dioxide ($\hbox {CO}_{2}$) sequestration consisting of seven species dissolved in water: $\hbox {CaCO}_{3}$, $\hbox {Ca}^{2+}$, $\hbox {CO}_{3}^{2-}$, $\hbox {H}^{+}$, $\hbox {OH}^{-}$, $\hbox {H}_2\hbox {CO}_{3}$, $\hbox{HCO}^{-}_{3}$ that interact through the following four equilibrium reactions:

5 $$\begin{aligned} \hbox {CaCO}_3&\rightleftharpoons \hbox {Ca}^{2+} + \hbox {CO}_{3}^{2-} , \end{aligned}$$

6 $$\begin{aligned} \hbox {HCO}^{-}_{3}&\rightleftharpoons \hbox {CO}_{3}^{2-} + \hbox {H}^{+} , \end{aligned}$$

7 $$\begin{aligned} \hbox {H}_{2}\hbox {CO}_{3}&\rightleftharpoons \hbox {CO}_{3}^{2-} + 2 \hbox{H}^{+} , \end{aligned}$$

8 $$\begin{aligned} \hbox {H}^{+} + \hbox {OH}^{-}&\rightleftharpoons \hbox {H}_{2}\hbox{O}. \end{aligned}$$

If we denote concentrations by square brackets, changes in concentrations (time-derivatives) by, e.g., $[\hbox {CaCO}_{3}]^{\prime }$, and rates $R_{1}, \ldots , R_{4}$ for the four reactions (positive in the leftward direction), the evolution of all concentrations can be solved from

9 $$\begin{aligned} \left[ \hbox {CaCO}_{3} \right] ^{\prime }= & {} - R_1, \end{aligned}$$

10 $$\begin{aligned} \left[ \hbox {HCO}^{-}_{3} \right] ^{\prime }= & {} -R_2, \end{aligned}$$

11 $$\begin{aligned} \left[ \hbox {H}_2\hbox {CO}_{3} \right] ^{\prime }= & {} - R_3, \end{aligned}$$

12 $$\begin{aligned} \left[ \hbox {OH}^{-} \right] ^{\prime }= & {} - R_4, \end{aligned}$$

13 $$\begin{aligned} \left[ {\mathrm {tot}} (\hbox {H}) \right] ^{\prime }= & {} \left( [\hbox {H}^{+}]+ [\hbox {HCO}^{-}_{3}]+2[\hbox {H}_2\hbox {CO}_{3}] - [\hbox {OH}^{-}]\right) ^{\prime } =0, \end{aligned}$$

14 $$\begin{aligned} \left[ {\mathrm {tot}} (\hbox {Ca}) \right] ^{\prime }= & {} \left( [\hbox {Ca}^{2+}]+[\hbox {CaCO}_{3}] \right) ^{\prime } = 0, \end{aligned}$$

15 $$\begin{aligned} \left[ {\mathrm {tot}} (\hbox {CO}_{3}) \right] ^{\prime }= & {} \left( [\hbox {CO}_{3}^{2-}]+ [\hbox {CaCO}_{3}] + [\hbox {HCO}^{-}_{3}] + [\hbox {H}_2\hbox {CO}_{3}] \right) ^{\prime } = 0. \end{aligned}$$

The first four equations define the primary species $\hbox {CaCO}_{3}$ , $\hbox {HCO}^{-}_{3}$, $\hbox {H}_2\hbox {CO}_{3}$, $\hbox {OH}^{-}$, while the last three equations involve the secondary species $\hbox {H}^{+}$, $\hbox {Ca}^{2+}$, $\hbox {CO}_{3}^{2-}$, as well as defining the (conservation of) *total* concentrations of those elements across all species. Following common notations^18^ and writing $\Psi _{j=1, \ldots , 3}$ for the total concentrations, $C_{j=1, \ldots , 3}$ for the secondary species, and $C_{i=1, \ldots , 4}$ for the primary species, Eqs. (13)–(15) can be written succinctly in terms of the *stoichiometry* coefficients $\nu _{ij}$ as

16 $$\begin{aligned} \Psi _{j} = C_{j} + \sum _{i=1}^{4} \nu _{ij} C_{i}, \quad \quad \nu _{ij} = \left( \begin{array}{cccc} 0 &{} 1 &{} 2 &{} -1\\ 1 &{} 0 &{} 0 &{} 0 \\ 1 &{} 1 &{} 1 &{} 0 \end{array} \right) . \end{aligned}$$

From the definitions Eqs. (13)–(15) it is clear that the total concentrations (or ‘total components’) are conserved in the reacting system and thus a natural choice as primary variables in the molar conservation Eq. (1) for species transport. More generally, all problems of interest involve water itself and we usually choose ${\mathrm {tot}} (\hbox {H})$ and ${\mathrm {tot}} (\hbox {O})$ as two of the total concentrations. We will refer to the number of total or primary components that need to be transported as $n_{p}$ and note that those are, in a sense, ‘bookkeeping’ quantities, whereas we will continue to use $n_{c}$ for the total number of actual molecular species in the mixture.

The different symbols $c_{i}$ versus $C_{i}$ refer to different unit systems: Phreeqc typically expresses all concentrations per kilogram or liter of water, whereas Eq. (1) involves intrinsic molar densities ($[{\mathrm {mol}}/{\mathrm {m}}^{3}]$). In coupling the transport and geochemistry, a unit conversion is made between *Osures* and Phreeqc that involves the (temperature, pressure, and composition dependent) aqueous phase mass density as computed from the CPA EOS^38^ (equivalently, PhreeqcRM can be provided with $[{\mathrm {mol}}/\mathrm {l}]$ concentrations together with a mass density).

Just as in most other reactive transport codes, a (sequential non-iterative) operator splitting approach is adopted in which the flow-transport problem is solved first without considering reactions, followed by the equivalent of a batch reaction calculation for each grid-cell (or node in the case of higher-order methods). More implementation details are provided below.

### Diffusion of chemical species

Molecular diffusion, as defined in irreversible thermodynamics, is driven fundamentally by gradients in chemical potentials. Under the assumptions of negligible temperature and pressure diffusion an expression is obtained in terms of gradients in compositions, which is the commonly used generalized Fick’s law. Thus, while total concentrations, as the conserved quantity, are a suitable choice for advective transport they are not natural variables for the diffusive flux^45^. The following equations are therefore for the $n_{c}$ physical species.

Diffusion of particles through a porous medium is affected by the geometry and connectivity of the pore network, and is different from diffusion in open space. The longer pathways in a porous medium are represented empirically in Eq. (2) by the factor $f(\phi ,\tau )\ [\cdot ]$, which is a function of porosity and tortuosity $\tau \ [\cdot ]$. The simplest option is $f(\phi ,\tau )=\phi$.

Both molecular diffusion and mechanical dispersion are considered, e.g., $\vec {J}_{i,\alpha } = \vec {J}_{\alpha , i}^{\mathrm {diff}} + \vec {J}_{\alpha , i}^{{\mathrm {disp}}}$. Mechanical dispersion is computed from

17 $$\begin{aligned} \vec {J}_{\alpha , i}^{{\mathrm {disp}}} = - c_\alpha \sum _{k=1}^{n_c -1} \vec {D}^{\mathrm {disp}}_{\alpha }\ \nabla x_{\alpha , k}, \end{aligned}$$

with the coefficients given by the tensor

18 $$\begin{aligned}&\vec {D}^{\mathrm {disp}}_{\alpha } = d_{t,\alpha } | \vec {u}_{\alpha } | \vec {I} + (d_{l,\alpha } - d_{t,\alpha }) \frac{\vec {u}_{\alpha } \vec {u}^{T}_{\alpha }}{|\vec {u}_{\alpha }|}, \end{aligned}$$

with $d_{l,\alpha }\ [{\mathrm {m}}]$ and $d_{t,\alpha }\ [{\mathrm {m}}]$ the longitudinal and transverse phase dispersivities, respectively, and $\vec {I}$ the identity matrix. There are only $n_{c}-1$ independent equations because by definition $\sum _{i} J_{i} = 0$, such that $J_{n_{c}} = - \sum _{i=1}^{n_{c}-1} J_{i}$. The $n_{c}-1$ equations for Fickian molecular diffusion are:

19 $$\begin{aligned} \vec {J}_{\alpha , i}^{\mathrm {diff}} = - c_\alpha \sum _{k=1}^{n_c -1} D^{\mathrm {Fick}}_{\alpha , ik}\ \nabla x_{\alpha , k}, \end{aligned}$$

with $x_{\alpha , i}\ [\cdot ]$ the phase compositions (molar fractions) and $D^{\mathrm {Fick}}_{\alpha , ik}\ [{\mathrm {m}}^{2}/{\mathrm {s}}]$ a full matrix of composition-dependent diffusion coefficients as derived from irreversible thermodynamics^30,39,46^.

It can easily be shown^47^ that only considering diagonal (‘self’) diffusion coefficients violates molar balance, because the commonly used $J_{i} \sim -D_{i} \nabla x_{i}$ cannot simultaneously satisfy $\sum _{i} J_{i} = 0$ and $\sum _{i} x_{i} = 1$. Specifically, for $n_{c}$ species we have $\sum _{i=1}^{n_c} x_{i } = 1$, which means that $\sum _{i=1}^{n_c}\nabla x_{i} = 0$. In other words, the compositional gradients are not all independent and one can be expressed in terms of the others. Choosing the last component, for instance, we have

20 $$\begin{aligned} \nabla x_{n_{c}} = - \sum _{i=1}^{n_c-1}\nabla x_{i} . \end{aligned}$$

The diffusive fluxes are also not all independent because, by definition (i.e., diffusion being the *deviation* of individual species fluxes from the average advective flux) $\sum _{i=1}^{n_c} J_{i} = 0$. Similar to Eq. (20), we choose to express the diffusive flux of the last component in terms of the other fluxes $J_{n_{c}} = - D_{c} \nabla x_{c} = - \sum _{i=1}^{n_c-1} J_{i} = - \sum _{i=1}^{n_c-1} D_{i} \nabla x_{i}$. Inserting $\nabla x_{n_{c}}$ from Eq. (20) that requires

21 $$\begin{aligned} \sum _{i=1}^{n_c - 1} (D_{n_c} - D_{i}) \nabla x_{i} = 0. \end{aligned}$$

For Eq. (21) to be true for any composition $x_{i}$ requires all $D_{i}= D_{n_{c}}$, i.e. all diagonal diffusion coefficients have to be the same. In other words, molar conservation is only guaranteed either for a single scalar diffusion coefficient for all components (which is not justified by experimental data) or requires a full matrix of multicomponent diffusion coefficients.

In terms of implementation, for diffusion problems Phreeqc is instructed to output not only the $n_{p}$ concentrations $\Psi _{j}$ but also the $n_{c}$ concentrations $C_{i}$ and $C_{j}$ (this requires more memory, but not more computational effort). Eq. (19) is then updated for each ‘real’ species across each grid face in the domain, and the contributions to the molar densities of $n_{p}$ total components follows from the stoichiometry (using Eq. (16)). An operator splitting step is used in the implementation: first the diffusive fluxes are computed as described, then, in updating Eq. (1) the divergence of the diffusive flux is essentially treated as a sink-source term of the total number of moles of $c_{i}$ entering or leaving the grid cell through all its faces in a given time-step.

### Nernst–Planck electromigration

Electrochemical migration refers to electrostatic forces coupling to charged particles that diffuse at different rates, which causes charge imbalance. Electric fields can force charged particles to diffuse when there are no compositional gradients or even diffuse from low to high concentrations, due to interaction with other species. Similar effects have been observed even in charge-neutral non-ideal mixtures such as hydrocarbon fluids^48^. Because the flux of one species can depend on the compositional gradients in all other species, this is another reason that a full matrix of diffusion coefficients is required.

The following expression has been used to model both Fickian ($J^{{\mathrm {Fick}}}_{i}$) and electrochemical ($J^{\mathrm {EK}}_{i}$) diffusion in the absence of externally induced currents and advective fluxes^49^:

22 $$\begin{aligned} J_{i} = J^{{\mathrm {Fick}}}_{i} + J^{\mathrm {EK}}_{i} = - D_{i} \nabla C_{i} + D_{i} C_{i} q_{i} \frac{\sum _{k} D_{k} q_{k} \nabla C_{k} }{\sum _{k} D_{k} q^2_{k} C_{k}} \end{aligned}$$

with $q_{k}$ the species charge, $C_{i}$ concentrations, and summations over all *dissolved* species. Eq. (22) is a simplified form of the Nernst-Planck equation.

To be consistent with the molar balance equation (1) and allowing for variable aqueous densities (compressibility), Eq. (22) is written in terms of aqueous phase molar density *c* and molar fractions $x_{i}=c_{i}/c$, similar to Eq. (19), as

23 $$\begin{aligned} J_{i} = - c D_{i} \nabla x_{i} + D_{i} x_{i} q_{i} \frac{\sum _{k} c D_{k} q_{k} \nabla x_{k} }{\sum _{k} D_{k} q^2_{k} x_{k}}, \end{aligned}$$

which assumes that diffusion coefficients have already been corrected for porosity and tortuosity effects.

As discussed in the previous section, this type of relation for diffusion in multicomponent mixtures is physically inconsistent. However it can be a reasonable approximation (when off-diagonal diffusion coefficients are small) and is implemented in this work as an option to allow comparisons to other reactive transport codes that rely on this formulation.

### Implementation

The numerical implementation of the mathematical framework described in the previous sections relies heavily on operator splitting, which permits choosing the most suitable numerical method for each subproblem. First, diffusive fluxes (Eqs. (17)–(19)) are computed using compositions, molar densities, and advective fluxes from the previous time-step. Second, the flow problem Eqs. (3)–(4) is simultaneously solved for pressures and fluxes by the implicit MHFE method. Third, the transport equations (Eqs. (1)–(2)) are updated by the DG method, using the previously computed diffusive fluxes. Other than the interpretation of total components (Eq. (16)) and the implementation of the Nernst-Planck Eq. (23) for electromigration, the implementation is identical to prior (non-reactive) works^20,32,33^, and is thus not repeated here in further detail.

After the transport equations have been updated for all components, PhreeqcRM is invoked to update the geochemistry. The geochemistry computations alter the compositions of reactive species, which is indicated by the $F^{\mathrm {react}}_i$ term in Eq. (1). As discussed above, PhreeqcRM is requested to output both the total component concentrations that are advected in Eq. (1) as well as all the physical species concentrations that are used to compute the diffusive fluxes (Eqs. (17)–(19)). The diffusive flux contributions of each species to the total component transport is derived using the stoichiometry as in Eq. (16).

The full reactive transport step is followed by an EOS-based update of fluid properties (molar and mass densities, compressibility, viscosity), as well as rock properties (porosity, permeability, fracture apertures) when dissolution and precipitation reactions are considered. For multiphase problems this would also involve phase stability and phase split computations that are iteratively coupled to the PhreeqcRM geochemistry update.

Explicit, implicit, and adaptive implicit Euler time-discretizations have been implemented, where the adaptive method uses an implicit update for grid cells that have a small Courant-Friedrichs-Lewy (CFL) time-step constraint^50^ and an explicit update elsewhere^33^. The advantage of implicit methods is that they are unconditionally stable and thus allow for larger time-steps. However, implicit methods are also known to exhibit excessive numerical dispersion. Moreover, (1) larger time-steps imply bigger changes in concentrations, which results in numerical convergence issues for PhreeqcRM, and (2) rock-fluid interactions and kinetic reactions are quite sensitive to time-step sizes. For these reasons, unless a fully coupled approach is used, an explicit transport update appears to provide the most accurate results (smaller time-steps also reduce the decoupling errors inherent to any operator splitting approach). The cost of using relatively small time-steps can be alleviated by (1) faster convergence of the non-linear geochemistry (similar to phase-split computations), and (2) the more trivial parallelization of an element-wise explicit transport and geochemistry update. The numerical examples, presented next, therefore all rely on the common implicit-pressure-explicit-composition (IMPEC) scheme.

## Numerical experiments

This section provides validation tests of the new model presented in this work by modeling three benchmark studies^6,49,51^. These examples cover a range of aqueous equilibrium reactions, tracer transport, isotope fractionation, electrochemical migration, Fickian diffusion, mechanical dispersion, and fluid-rock cation exchange reactions. Additional numerical experiments illustrate the improved features in this formulation.

### Example 1: transient electromigration benchmark

This benchmark problem^49,51^ consideres a mixture of $\hbox {H}^{+}$, $\hbox {NO}_3^{-}$, $\hbox {Na}^{+}$, $\hbox {Cl}^{-}$ (primary), and $\hbox {OH}^{-}$ (secondary) components in a small $100\times 100\mu {\mathrm {m}}$ element 1D grid with a constant composition Dirichlet boundary condition (BC) on the left and no-flow Neumann BC on the right boundary. $\hbox {Na}^{+}$ and $\hbox {Cl}^{-}$ BC and initial conditions are the same (0.1 mM), but $\hbox {NO}_{3}^{-}$ ($10^{-3}$ mM) and $\hbox {OH}^{-}$ ($10^{-7}$ mM) BC values are a hundred times lower than the initial domain concentrations, while the pH on the boundary and inside the domain are 6 and 4, respectively. Nernst-Planck diffusion is modeled, without advection, for a period of one hour. Further details are provided in the comparative benchmark study^49^.

The physics of this problem is that $\hbox {H}^{+}$ and $\hbox {NO}_{3}^{-}$ diffuse towards (and out of) the left boundary where their concentrations are significantly lower. However, because the diffusion coefficient for $\hbox {H}^{+}$ ($9.31\times 10^{-9}\ {\mathrm {m}}^{2}/{\mathrm {s}}$) is about five times higher than that of $\hbox {NO}_{3}^{-}$ ($1.9\times 10^{-9}\ {\mathrm {m}}^{2}/{\mathrm {s}}$) and $\hbox {H}^{+}$ leaves through the left boundary more rapidly, this sets up electrochemical migration of $\hbox {Na}^{+}$, $\hbox {Cl}^{-}$ to maintain charge balance, even though there is no initial gradient in the compositions of those species. Fick’s law does not capture this effect, which would result in violating electroneutrality.

**Figure 1:**
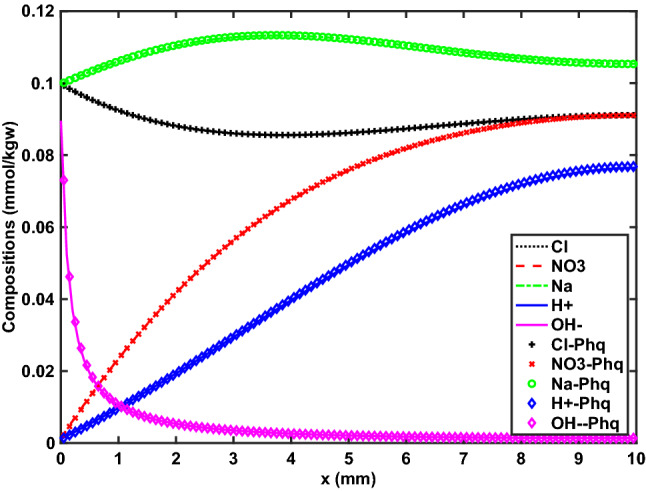
Example 1. Concentrations of $\hbox {H}^{+}$, $\hbox {NO}_{3}^{-}$, $\hbox {Na}^{+}$, $\hbox {Cl}^{-}$, and $\hbox {OH}^{-}$ (the latter $\times 10^{4}$) throughout 1 cm domain after one hour. Computed with Phreeqc (symbols) and *Osures* (solid lines).

The original benchmark study^49^ compared results from Phreeqc, CrunchFlow, and MIN3P, finding good agreement. Figure 1 compares Phreeqc results to those from the new reactive transport simulator presented in this work, *Osures*, demonstrating that we can match this benchmark problem perfectly when using the same lowest-order approximation (FV).

**Figure 2:**
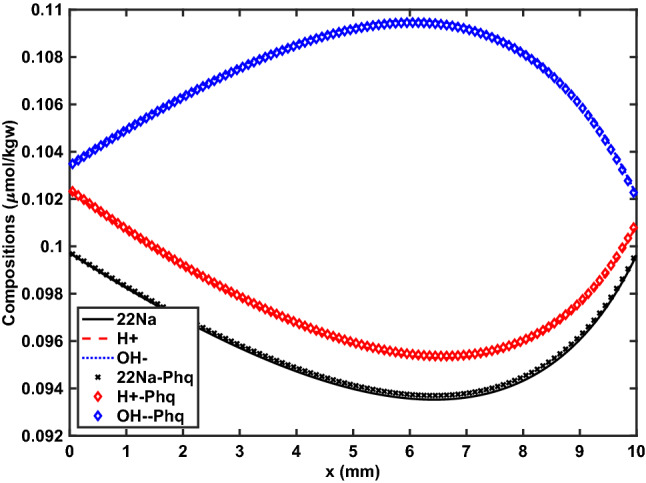
Example 2. Steady state concentration (after 24 hrs) of $^{22}\hbox {Na}^{+}$ ($\times 10^{-2}$), $\hbox {H}^+$, and $\hbox {OH}^{-}$ computed with Phreeqc (symbols) and *Osures* (solid lines).

### Example 2: tracer isotope diffusion

This benchmark problem has a similar set-up as the previous example, but with $\hbox {NO}_{3}^{-}$ replaced by $^{22}\hbox {Na}^{+}$, which is treated as a separate species with the same diffusion coefficient as $\hbox {Na}^{+}$ ($1.33\times 10^{-9}\ {\mathrm {m}}^{2}/{\mathrm {s}}$). Both left and right boundaries now have a constant concentration Dirichlet BC, which is the same for pH (7), $^{22}\hbox {Na}^{+}$ ($10^{-6}$ nM), and $\hbox {OH}^{-}$ ($10^{-4}$ nM), but five times higher for $\hbox {Na}^{+}$ and $\hbox {Cl}^{-}$ on the left boundary (0.5 mM) than on the right boundary (0.1 mM). In other words, a fixed gradient in $\hbox {Na}^{+}$ and $\hbox {Cl}^{-}$ is imposed. The problem is modeled until a steady state is reached.

A more detailed discussion is provided in the literature^49^, but the key point is that the different diffusion coefficients (and fluxes) of each species again cause non-linear electrochemical coupling effects, which cause significant isotope fractionation for $^{22}\hbox {Na}^{+}$ even though its concentrations are fixed at the same value on the boundaries. This effect is demonstrated in Fig. 2, which also shows excellent agreement with Phreeqc simulation results (modeled again with a lowest-order discretization).

**Figure 3:**
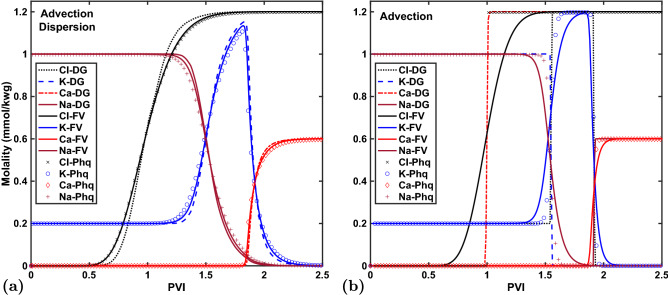
Example 3. Concentrations of potassium, sodium, chloride, and calcite in effluent of 8 cm long cation exchange column during 2.5 PV of flooding. Computed with Phreeqc (symbols) and *Osures* (solid lines) on a 40 element 1D grid with (**a**) and without (**b**) mechanical dispersion. Higher-order DG results are computed on the same 40 element grid for the advection-dispersion simulation but on a finer 400 element grid for the advection-only case to match its numerical dispersion free Phreeqc results.

**Figure 4:**
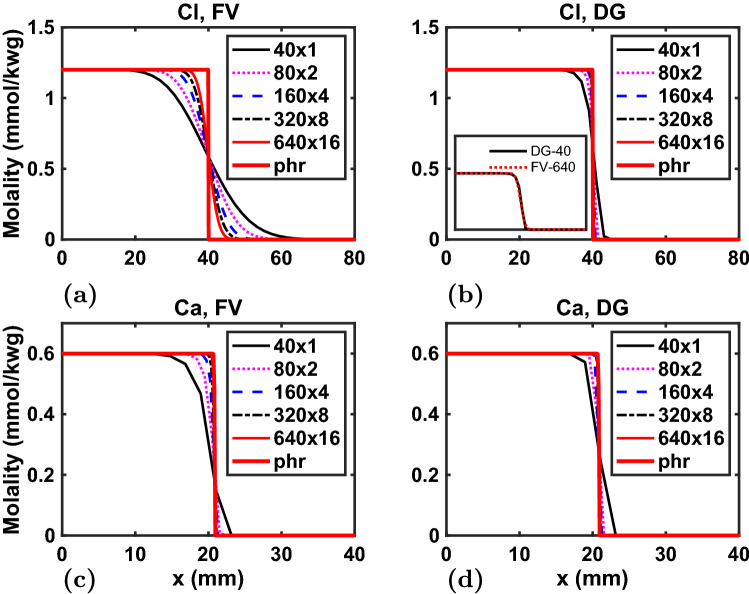
Example 4. Concentration profiles at 50% PVI of $\hbox {Cl}^{-}$ (**a**, **b**) and $\hbox {Ca}^{2+}$ (**c**, **d**) from FV (**a**, **c**) and DG (**b**, **d**) simulations on $40\times 1$, $80\times 2$, $160\times 4$, $320\times 8$, and $640\times 16$ grids. Phreeqc (phr) results are for a 640 element 1D grid. The inset compares $640\times 16$ FV and $40\times 1$ DG simulations. For clarity, only the left half of the column is shown for $\hbox {Ca}^{2+}$.

### Example 3: advection-dispersion transport and cation exchange

This third benchmark is Example 11 in the Phreeqc 3.0 manual^6^, which has been modeled extensively with multiple reactive transport models that use the Phreeqc geochemical engine, such as PHREEQM-2D, UTCOMP-PhreeqcRM, and PHT3D^3,35,52–54^. 2.5 pore volumes (PV) of a calcium-chloride solution (with 0.6 mM $\hbox {Ca}$ and 1.2 mM $\hbox {Cl}$) are injected into a 8 cm long cation exchange column (discretized by a 40 element 1D grid) that is initially saturated with a sodium-potassium-nitrate solution (1 mM $\hbox {Na}$, 0.2 mM $\hbox {K}$, and 1.2 mM $\hbox {NO}_{3}^{-}$) in equilibrium with an exchanger with 1.1 mM capacity.

The complexities in this example, as compared to the previous ones, are the rock-fluid reactions as well as velocity dependent mechanical dispersion (Eq. (18)).

The physics of the problem is that $\hbox {Cl}^{-}$ is a conservative tracer (arriving at the outlet after one pore volume injected (PVI) in the absence of dispersion) while the injected $\hbox {Ca}^{2+}$ exchanges with the $\hbox {Na}^{+}$ in the column until it is used up (around 1.5 PVI). The potassium is released later ($\sim 1.75$ PVI) because it is a stronger exchanger (larger $\log K$). Only after all $\hbox {Na}^{+}$ and $\hbox {K}^{+}$ have been released does $\hbox {Ca}^{2+}$ show up in the effluent at its injected concentration. Further discussion, including comparison to an analytical solution for the $\hbox {Cl}^{-}$ breakthrough curve, is provided in the literature^6^.

**Figure 5:**
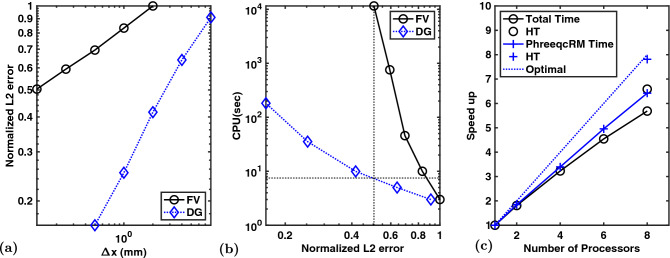
Example 4. $\hbox {L}_2$ errors of FV and DG simulations on 7 levels of grid refinement. Errors (**a**) are normalized by the largest $\hbox {L}_2$-norm, which is for a FV simulation on a $40\times 1$ element grid. Given the different error ranges for FV versus DG (note the log-log scales), the former are shown for the 5 finest grids (starting with 40 elements) and the latter for the 5 coarsest grids (starting from 10 elements). The computational cost of all simulations is plotted (logarithmically) versus the corresponding numerical error (**b**). OpenMP parallel scaling is shown for up to 8 cores/threads and one example of 16 threads on 8 cores with hyper-threading (HT) (**c**).

**Figure 6:**
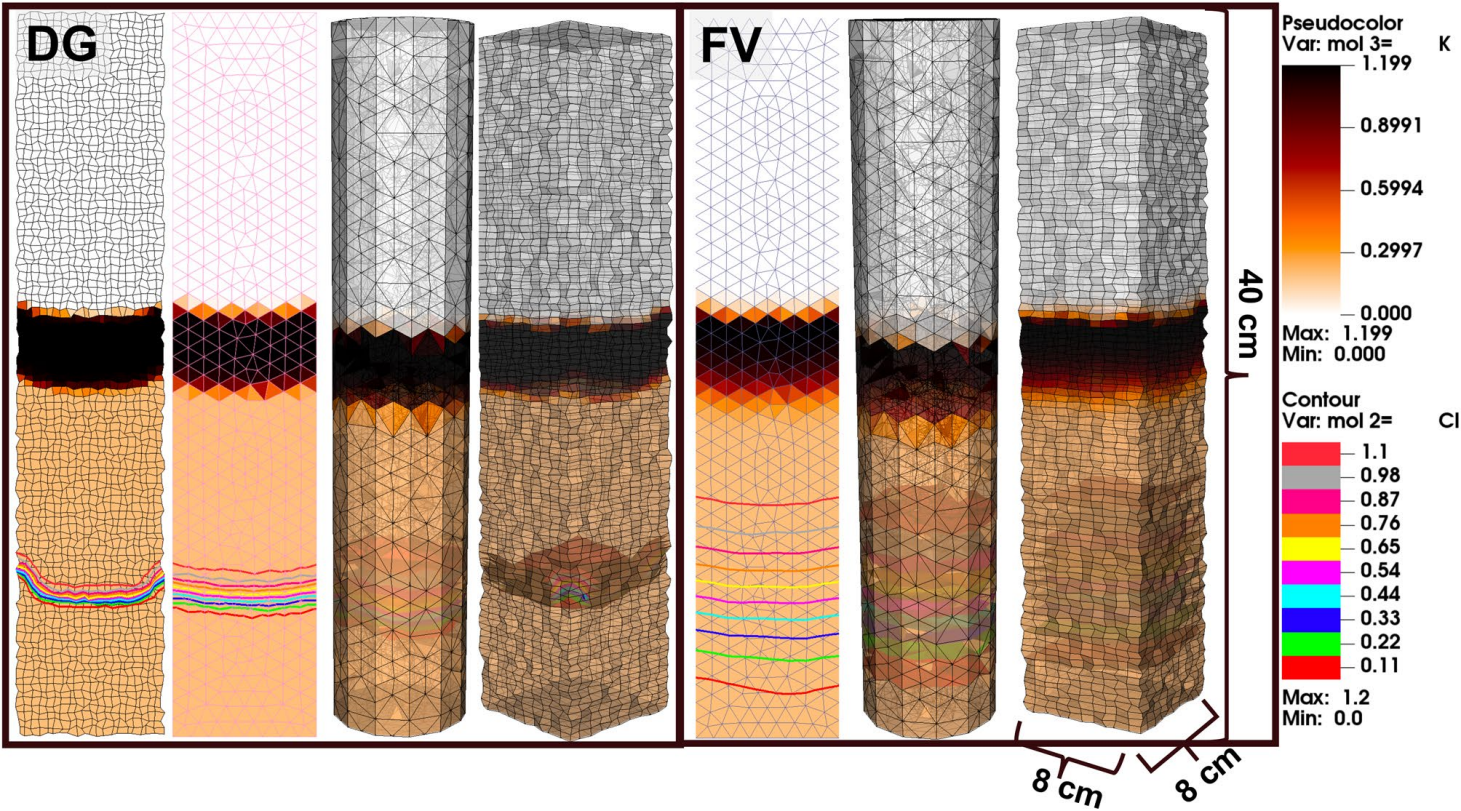
Example 5. Potassium and chloride concentrations after 80% PVI for cation exchange simulations on 2D and 3D unstructured grids.

**Figure 7:**
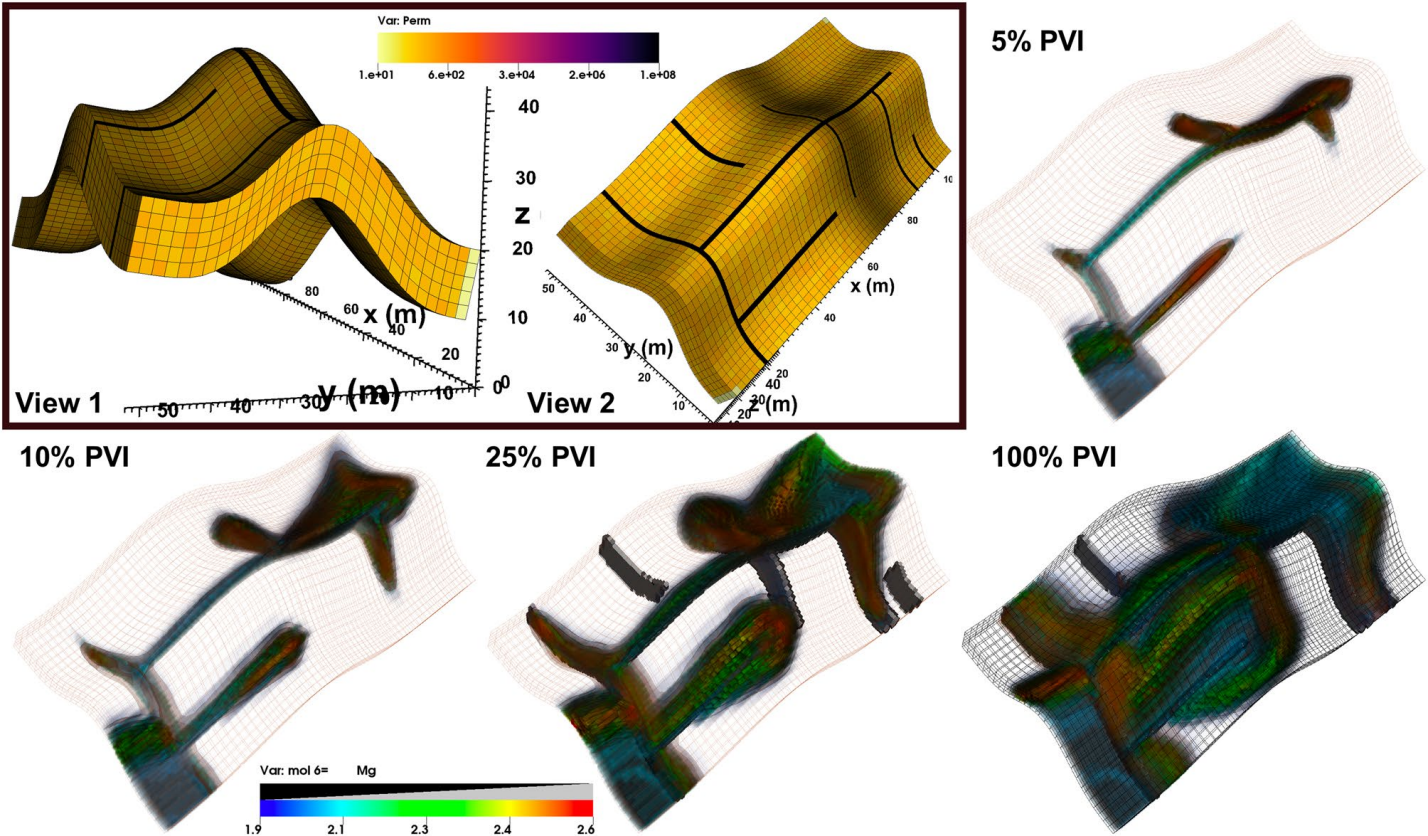
Example 6. Magnesium concentrations after 5%, 10%, 25%, and 100% PVI in a discretely fractured formation. The fractures, permeability field, and domain geometry are illustrated in the inset. Fracture locations are also shown in the 25% and 100% PVI panels.

Figure 3 shows that the *Osures* results agree well with Phreeqc. Differences are entirely due to varying degrees of numerical dispersion. The *Osures* FV simulations closely match the advection-dispersion Phreeqc results (left), which also uses a lowest-order transport update. DG simulations show less numerical dispersion than both the *Osures* and Phreeqc FV results. For advection-only, Phreeqc simply *shifts* concentrations from one grid cell to the next, which does not involve an actual discretized transport equation and does not result in significant numerical dispersion (though the profiles do exhibit slightly spread-out fronts that sharpen on finer grids). The *Osures* FV simulation exhibits considerably more numerical dispersion. A higher-order DG simulation on a 400 element grid is shown to eliminate the numerical dispersion and match the Phreeqc data. A more detailed analysis of these differences in accuracy, and associated numerical dispersion, are presented in the next example.

With these three benchmark examples providing confidence that Phreeqc was successfully coupled to a new FE flow and transport framework, the next examples demonstrate the novel and powerful features that this approach provide.

### Example 4: higher-order DG for reactive transport, convergence analyses, and parallelization

The majority of reactive transport codes rely on lowest-order methods for flow and transport. Earlier work^35^ found that a higher-order total variation diminishing (TVD) approach resulted in unphysical oscillations in concentrations and argued that this might be inherent to higher-order modeling of reactive transport, though PHT3D^3^ also has a TVD option. In this work, both first and second order Discontinuous Galerkin methods are adopted, with the former being equivalent to a FV approach (with element-wise-constant properties and an upwind numerical flux). In this example, we clearly demonstrate the power of these higher-order methods in producing accurate results on coarser grids, and achieving high computational efficiency.

We consider the same cation exchange problem without *physical* dispersion to focus on the *numerical* dispersion that is an artifact of discretization errors. FV and DG simulations are performed on 7 levels of grid refinement: $40\times 1$, $80\times 2$, $160\times 4$, $320\times 8$, $640\times 16$, and two coarser $10\times 1$, $20\times 1$ grids (the latter 2 for DG only). The Phreeqc simulation is redone on a 640 element 1D grid to serve as the ‘true’ solution of perfect piston-like step-functions to compare all others to.

Figure 4 presents spatial concentration profiles at 50% PVI of the $\hbox {Cl}^{-}$ tracer and $\hbox {Ca}^{2+}$, which exchanges with $\hbox {Na}^{+}$ in the column and exhibits a delayed front. Qualitatively, it is clear that the FV simulations are considerably more dispersed than for DG. The inset in Fig. 4 demonstrates that the FV simulation on the finest $640\times 16$ grid has the same numerical dispersion as the coarsest $40\times 1$ DG simulation and have still not converged to the Phreeqc profile. Interestingly, the $\hbox {Ca}^{2+}$ profiles, which are a result of both advective transport and geochemical rock-fluid reactions, are less dispersed.

To demonstrate the advances that higher-order methods can bring to reactive transport modeling more clearly, Fig. 5 explores the numerical errors, specifically the $\hbox {L}_2$ norms, more quantitatively. A log-log plot of numerical errors versus characteristic grid sizes ($\Delta x$) for all simulation shows the *convergence rate* of each method. The convergence rate (slope) for DG computed through this numerical experiment is 2.5 higher than that for the FV simulations, i.e. even more than the factor 2 expected from theory (linear versus quadratic convergence).

Why is this important? That is illustrated in Fig. 5b for the computational costs (CPU time) of all simulations versus their accuracy (as expressed by the $\hbox {L}_{2}$ norms). The figure makes the critical point that even though a higher-order method requires more CPU time *on a given grid* it allows for far coarser grids than a lower-order methods to achieve the same accuracy. The dotted lines illustrates how a 7.5 second DG simulation can achieve the same accuracy as a 3.2 hour FV simulation on a much finer grid, i.e. a three orders of magnitude improvement in computational efficiency (the same degree of speed-up was found in a similar analysis for multiphase multicomponent gas-oil simulations in an earlier work^21^). Given the notoriously high computational cost of reactive transport simulations, this is a major advancement in the state-of-the-art.

Figure 5c also shows decent (though not optimal) parallel scaling of our new reactive transport model, using the OpenMP shared-memory capabilities of PhreeqRM on an 8-core Intel Core i9 processor. The scaling analysis is made somewhat non-trivial by Intel’s use of variable clock speeds (Turbo Boost), ranging from 2.4 GHz to 4.4 GHz for this processor, depending on load and associated internal temperatures; hyper-threading provides another $\sim$ 20% improvement when using 16 threads, achieving optimal scaling of the geochemistry. A important advantage of our DG transport update is that it is local, i.e., each grid cell is updated independently, making it trivially parallelizable. The MHFE method involves a global pressure solve, which is currently not effectively parallelized and results in a weaker scaling of the full simulator. Distributed memory parallelization (MPI) for larger scale problems on cluster environments is a work in progress.

### Example 5: unstructured triangular, quadrilateral, hexahedral, and tetrahedral grids

Apart from the low numerical dispersion and parallelizable features of the DG transport, perhaps the most obvious advantage of FE methods is that they are a natural choice for problems that benefit from unstructured grids. To demonstrate the robustness of our proposed MHFE-DG methods for reactive transport on unstructured grids, we model the same cation exchange problem on 4 different grid types: triangular (770 elements grid), tetrahedral (15,894 element cylinder), and poor-quality (for demonstration purposes) quadrilateral ($20\times 100$) and hexahedral ($10\times 10 \times 100$) grids with physical dimensions of 8 cm ($\times 8$ cm) $\times 40$ cm. The potassium (semi-transparent colors) and chloride (contours/surfaces) concentrations are shown in Fig. 6 after 80% PVI for both DG and FV simulations, with the latter again exhibiting far more numerical dispersion. Otherwise, the simulation results on all grid types are the same.

This example simply validates the generalization of Phreeqc capabilities to a higher-order FE approach on any type of 2D and 3D unstructured grids. Unstructured grids allow one to honor the true geometry of both laboratory scale problems, such as heterogeneous and perhaps fractured cylindrical core samples, as well as complex geological formations. Truly unstructured grids (e.g., tetrahedral) can also avoid the many severely pinched elements and dead cells that can plague the logically cartesian corner–point grids that are commonly used in industry simulators to accommodate legacy finite difference formulations.

### Example 6: discrete fractures

As a last example, we consider the most complicated problem of reactive transport with rock-fluid interactions in an unstructured 3D domain with multiple connected and disconnected discrete fractures. The domain represents a $100\times 50\times 10\ {\mathrm {m}}^{3}$ deposition that was deformed over geological time into an anticlinal structure and subsequently tectonically fractured. The matrix permeability has a log-normal distribution between 50 and 200 md. and 25% porosity. Five connected and two disconnected discrete fractures each have an aperture of 1 mm. and permeability of $10^{8}$ md. The domain is discretized by 9,150 irregular hexahedra, as illustrated in Fig. 7.

In terms or reactive transport, we consider the mixing of two South American waters (IW1 and PW1) with compositions and other properties described in the literature^35,55^. IW1 is injected uniformly from a perforated vertical well at the lower-left corner and water is produced from the diagonally opposite corner. The waters contain 26 dissolved species that interact through the following equilibrium reactions:

24 $$\begin{aligned} \hbox {H}_{2}\hbox {O}&\rightleftharpoons \hbox {H}^{+} + \hbox {OH}^{-}, \end{aligned}$$

25 $$\begin{aligned} \hbox {CO}_{3}^{2-} + \hbox {H}^{+}&\rightleftharpoons \hbox {HCO}_{3}^{-}, \end{aligned}$$

26 $$\begin{aligned} \hbox {Na}^{+} + \hbox {CO}_{3}^{2-}&\rightleftharpoons \hbox {NaCO}_{3}^{-}, \end{aligned}$$

27 $$\begin{aligned} \hbox {Na}^{+} + \hbox {H}^{+} + \hbox {CO}_{3}^{2-}&\rightleftharpoons \hbox {NaHCO}_{3}, \end{aligned}$$

28 $$\begin{aligned} \hbox {Na}^{+} + \hbox {H}_{2}\hbox {O}&\rightleftharpoons \hbox {NaOH} + \hbox {H}^{+}, \end{aligned}$$

29 $$\begin{aligned} \hbox {Mg}^{2+} + \hbox {CO}_{3}^{2-}&\rightleftharpoons \hbox {MgCO}_{3}, \end{aligned}$$

30 $$\begin{aligned} \hbox {Mg}^{2+} + \hbox {H}^{+} + \hbox {CO}_{3}^{2-}&\rightleftharpoons \hbox {MgHCO}_{3}, \end{aligned}$$

31 $$\begin{aligned} \hbox {Mg}^{2+} + \hbox {H}_{2}\hbox {O}&\rightleftharpoons \hbox {MgOH}^{+} + \hbox {H}^{+}, \end{aligned}$$

32 $$\begin{aligned} \hbox {Ca}^{2+} + \hbox {CO}_{3}^{2-}&\rightleftharpoons \hbox {CaCO}_{3}, \end{aligned}$$

33 $$\begin{aligned} \hbox {Ca}^{2+} + \hbox {CO}_{3}^{2-} + \hbox {H}^{+}&\rightleftharpoons \hbox {CaHCO}_{3}^{+}, \end{aligned}$$

34 $$\begin{aligned} \hbox {Ca}^{2+} + \hbox {H}_{2}\hbox {O}&\rightleftharpoons \hbox {CaOH}^{+} + \hbox {H}^{+}, \end{aligned}$$

35 $$\begin{aligned} \hbox {Ba}^{2+} + \hbox {CO}_{3}^{2-}&\rightleftharpoons \hbox {BaCO}_{3}, \end{aligned}$$

36 $$\begin{aligned} \hbox {Ba}^{2+} + \hbox {CO}_{3}^{2-} + \hbox {H}^{+}&\rightleftharpoons \hbox {BaHCO}_{3}^{+}, \end{aligned}$$

37 $$\begin{aligned} \hbox {Ba}^{2+} + \hbox {H}_{2}\hbox {O}&\rightleftharpoons \hbox {BaOH}^{+} + \hbox {H}^{+}, \end{aligned}$$

38 $$\begin{aligned} \hbox {Sr}^{2+} + \hbox {CO}_{3}^{2-}&\rightleftharpoons \hbox {SrCO}_{3}, \end{aligned}$$

39 $$\begin{aligned} \hbox {Sr}^{2+} + \hbox {CO}_{3}^{2-} + \hbox {H}^{+}&\rightleftharpoons \hbox {SrHCO}_{3}^{+}, \end{aligned}$$

40 $$\begin{aligned} \hbox {Sr}^{2+} + \hbox {H}_{2}\hbox {O}&\rightleftharpoons \hbox {BaOH}^{+} + \hbox {H}^{+}. \end{aligned}$$

Moreover, the rock has a cation exchange capacitance of 1.1 mmol/kg and is initially at equilibrium with the initial PW1 water. The exchange reactions within the dissolved species are:

41 $$\begin{aligned} \hbox {Na}^{+}\hbox {X}^{-} + \hbox {H}^{+}&\rightleftharpoons \hbox {H}^{+}\hbox {X}^{-} + \hbox {Na}^{+} , \end{aligned}$$

42 $$\begin{aligned} 2 \hbox {Na}^{+}\hbox {X}^{-} + \hbox {Ca}^{2+}&\rightleftharpoons \hbox {Ca}^{+}\hbox {X}_{2}^{-} + \hbox {Na}^{+} , \end{aligned}$$

43 $$\begin{aligned} 2 \hbox {Na}^{+}\hbox {X}^{-} + \hbox {Mg}^{2+}&\rightleftharpoons \hbox {Mg}^{+}\hbox {X}_{2}^{-} + \hbox {Na}^{+}, \end{aligned}$$

44 $$\begin{aligned} 2 \hbox {Na}^{+}\hbox {X}^{-} + \hbox {Ba}^{2+}&\rightleftharpoons \hbox {Ba}^{+}\hbox {X}_{2}^{-} + \hbox {Na}^{+} , \end{aligned}$$

45 $$\begin{aligned} 2 \hbox {Na}^{+}\hbox {X}^{-} + \hbox {Sr}^{2+}&\rightleftharpoons \hbox {Sr}^{+}\hbox {X}_{2}^{-} + \hbox {Na}^{+} , \end{aligned}$$

46 $$\begin{aligned} \hbox {K}^{+}\hbox {X}^{-} + \hbox {H}^{+}&\rightleftharpoons \hbox {H}^{+}\hbox {X}^{-} + \hbox {K}^{+}, \end{aligned}$$

47 $$\begin{aligned} 2 \hbox {K}^{+}\hbox {X}^{-} + \hbox {Ca}^{2+}&\rightleftharpoons \hbox {Ca}^{+}\hbox {X}_{2}^{-} + \hbox {K}^{+}, \end{aligned}$$

48 $$\begin{aligned} 2 \hbox {K}^{+}\hbox {X}^{-} + \hbox {Mg}^{2+}&\rightleftharpoons \hbox {Mg}^{+}\hbox {X}_{2}^{-} + \hbox {K}^{+} , \end{aligned}$$

49 $$\begin{aligned} 2 \hbox {K}^{+}\hbox {X}^{-} + \hbox {Ba}^{2+}&\rightleftharpoons \hbox {Ba}^{+}\hbox {X}_{2}^{-} + \hbox {K}^{+} , \end{aligned}$$

50 $$\begin{aligned} 2 \hbox {K}^{+}\hbox {X}^{-} + \hbox {Sr}^{2}+&\rightleftharpoons \hbox {Sr}^{+}\hbox {X}_{2}^{-} + \hbox {K}^{+}. \end{aligned}$$

The concentrations of $\hbox {Ca}^{2+}$, $\hbox {Mg}^{2+}$, $\hbox {Ba}^{2+}$, $\hbox {Sr}^{2+}$ and $\hbox {HCO}_{3}^{-}$ are higher in the IW1 injection water than the initial PW1. As the two waters mix, the accumulation of metal cations drives many of the equilibrium reactions in the rightward direction. Isosurfaces of all the cations show similar trends, with magnesium concentrations shown in Fig. 7 at 5%, 10%, 25%, and 100% PVI as the two waters mix and cations exchange with the rock. Not surprisingly, fluid flow is highly channelized through the connected fractures with little effect from the disconnected ones, and the introduced water reaches the opposite side of the formation after only 10% PVI.

The chemistry of the problem is discussed in further detail in the cited references, which also present simulations on one-dimensional grids with CMOST and CMG-STARS^55^ and one and two-dimensional structured grids with UTCHEM-iPhreeqc^35^. The purposed of this example (and the previous) is to show that we can extend such reactive transport capabilities to unstructured 3D and discretely fractured grids, while significantly reducing numerical artifacts (grid sensitivity and numerical dispersion) by the use of higher-order FE methods. Using parallel capabilities on a 28-core cluster, the simulation completed in under an hour.

## Conclusions and future work

This work presents a first step in using advanced (Mixed Hybrid and Discontinuous Galerkin) higher-order FE methods to model reactive transport problems, particularly those that involve strong heterogeneities in rock properties (including discrete fractures) and non-trivial domain geometries, which benefit from unstructured gridding. The MHFE method is known to provide superior velocity fields on such grids^20,23,24^, while DG has the advantages of strict mass conservation at the element level, trivial parallelization, and low numerical dispersion^21,56^. To adopt these methods for reactive transport problems, a sequential coupling to the PhreeqcRM geochemistry engine was implemented.

Multiple benchmark problems from the literature were used to validate and compare this new modeling framework to a range of other reactive transport codes for problems involving both equilibrium and rock-fluid reactions, and for different transport mechanisms, such as advection, Fickian diffusion, Nernst-Planck diffusion, and mechanical dispersion. Another set of numerical experiments demonstrate advanced capabilities on unstructured triangular, quadrilateral, hexahedral, and tetrahedral grids with heterogeneous rock properties and connected and disconnected fractures. Perhaps most importantly, we demonstrate quantitatively how the higher-order convergence rate translates into computational efficiency improvements of up to three orders of magnitude, with another order of magnitude gain from parallelization on consumer grade shared-memory processors. Further efficiency gains may be achieved by not updating the geochemistry in grid cells where concentrations have not changed after a transport update (e.g., far away from an invading fluid front). The latter approach is also used successfully in avoiding costly phase-split computations in multiphase multicomponent flow problems^57^.

The first implementation of higher-order FE reactive transport modeling presented in this work only considers the rock solid and a single aqueous phase. Future work will extend this framework to allow for water, oil, and gas phases. For such multiphase problems, iterations will be required to guarantee thermodynamic equilibrium by matching the species chemical potentials (or fugacities) computed by Phreeqc for the aqueous phase to those from the full multiphase problem. The objective is to allow the use of accurate equations of state, such as cubic-plus-association^38^, that consider the self-association of polar water molecules as well as cross-association with molecules such as $\hbox {CO}_{2}$. The latter is of particular interest in the context of geological $\hbox {CO}_{2}$ sequestration^58^. Conversely, the effect of salinity on, e.g., $\hbox {CO}_{2}$ solubilities in water, pH changes, and $\hbox {CO}_{2}$-rock interactions requires consideration of aqueous geochemistry.

The development and adoption of reactive transport simulators, while matured significantly in recent years, is arguably in an earlier stage than the innumerable numerical methods and simulators for non-reactive flow and transport used in, e.g., hydrogeology and petroleum engineering. This work is intended to help further bridge that gap in marrying state-of-the-art geochemistry with modern FE reservoir simulation tools.
